# European Travel

**Published:** 1914-06

**Authors:** 


### European Travel.

It is intended to offer here, some general suggestions that may be of service to those younger members of the medical profession who are contemplating post graduate study and to those older ones who have in mind a vacation and who are more or less desirous of applying some part of it to medical study of a less formal nature. It is not intended to enter into the details of post graduate study and, as those unfamiliar with Europe usually magnify the economic and some other aspects of the problem, more attention will be given to these than may perhaps seem dignified.

In the first place, it is not entirely a matter of patriotic prejudice to state that except for some few lines of scientific work and for the massing together of certain rare forms of disease so that a general experience can be obtained rapidly, just as good post graduate study can be done in America as in Europe. It may be conceded that the average European practitioner is better trained and does better routine work than the average American, although whatever inequality may exist is rapidly passing away on account of the dying out of the men educated under former, deficient standards and the establishment of higher standards in American colleges. But, so far as the leaders of medical thought are concerned, and even those who follow in the first ranks of routine practice, it is now pretty generally conceded on both sides of the Atlantic that there is not much to choose. In many instances, both the individual man and the individual institution in America, are admitted to be superior. In equipment, the average American hospital stands far higher than most of those in Europe.

From the purely economic standpoint, it must also be recognized that the prestige of European study has declined as it has become more common. In fact, we have arrived at the point at which information and ability are judged rigorously by actual facts and not by appearances.

Granting that a recent American graduate must consider time and money closely, it is doubtful whether he should undertake European study until he has definitely decided upon some special line of interest. On the other hand, the longer he waits the more difficult and the less profitable does it become to lose established practice and to undertake a radical change of interests, based on a foreign post graduate course. Not to mention the fact that the man who feels that he must wait till he has amassed a comfortable capital from the proceeds of practice, usually finds that it is all he can do to keep even with daily demands. For a man of mature years, well established in any line of medical work, it is generally advisable to regard the European trip as a vacation, and to supplement it with such informal medical study at clinics or lectures, and such special courses, as may appeal to him.

Within recent years, the slogan has gone forth "See America first." Granted that one has had the usual amount of trips to a dozen or more American cities, he will usually find the remainder surprisingly lacking in special interest while, with some few exceptions, American small towns are uninteresting. hence, the slogan "See America first" holds good mainly as a matter of patriotic pride, of preference for scenery over historic and human interests, and on account of special interest in certain lines of scientific study, mainly coming under geologic, botanic, zoologic and archaelogic subdivisions. Generally speaking, American scenery is on larger scales than European, but otherwise not superior and for any one seeking thrills in history, and in differences of human life, Europe has a great advantage.

There is no use trying to dodge the fact that more Americans would undertake European travel, if it were not for a dread of great expense and of danger and inconvenience incident to the mere foreignness of the conditions. It is pardonable, therefore, to speak plainly on these matters. For any European trip, of whatever duration, one must count the passage. Roughly speaking, the "ante" is at least $100 for second cabin passage, plus the round trip fare and incidentals to and from the American sea port. For first cabin, the minimum round trip fare is about $150, plus the same preliminaries. For obvious reasons, it is strongly advised not to take a second cabin passage unless on a one-cabin ship. Aside from this inevitable expense, it may be estimated, approximately, that the various expenses of European travel will amount to about two-thirds of what they would, on the same scale in America. Of course, if one follows the beaten track of wealthy Americans, or if he makes his trip a round of entertainment and dissipation, he will not find this statement true. It is not only cheaper, but much interesting, to travel as Europeans do, to lodge at good hotels built for Europeans, to eat at native restaurants, to go among the people, to attend concerts and decent theatres and to visit museums and parks, rather than places where one would be ashamed to be seen at home.

As to safety, railroad and other accidents are less common in Europe than America. The system of registration at a hotel or boarding house, on slips returned to the Police Departments, practically insures against mysterious disappearances. Any one who keeps sober, behaves himself and keeps his wits about him, is probably a good deal safer anywhere in western Europe than in America. The average European is also brought up on translations or paraphrases of wild west literature and is just a trifle afraid of an American. But he also regards him as rolling in wealth, and it is self evident that one who displays his money, is ignorant of native languages and customs, and ventures alone into disreputable places or those distant from the crowd, renders himself liable to annoyance, extortion and violence.

Railroad fares. It should be remembered that in England the ordinary standard is third-class; on the continent, second class; corresponding to American first class coach service, and costing about 2 cents a mile. Third class, continental, is approximately equivalent to suburban trolley accommodations, costs about one and one-third cents a mile, and may be used for short trips comfortably enough. First class costs about 3 cents a mile, but, except for the gratification of pride, and the presence of tidies on the cushions, is really no better than second class. Isolated compartments are common enough in England and are found on short-distance trains on the continent. Otherwise, most cars are built with corridors, have toilet rooms, and for any class resemble a state room on an American parlor car. It is impossible to make exact comparisons between Europe and America, and there are great differences in different countries and different trains, but on the whole, it is evident that railroad travel is no more expensive in Europe than in America. Its accessories are mostly less expensive and the relative shortness of distance as to political divisions, changes of scenery, etc., make it seem much less expensive. To avoid fatigue, to obtain a bird's eye view of the country, and because sleeping car service is not quite so well developed as in America, it is strongly advised to travel only by day. Stop-overs are very freely given and one can usually have a choice of route, even a choice of transfer to boat, between any two division points. But, it should be remembered that European railroads are by no means so liberal in regard to baggage as American, and, both for economy and convenience, it is better to travel with a suit case if possible. At most places, porters can be found to carry baggage to hotels, for a small fee.

Hotels. The average good European hotel charges about 80 cents to a dollar for a room without bath; 30 cents for a very light breakfast; a dollar or a dollar and a half for table d'hote dinner. Very appetizing and apparently wholesome meals can be obtained almost anywhere, either at a fixed price or a la carte, at about half what they would cost in America, and up to the full American price, according to circumstances. Tipping is more frequent than in America, but the relative amounts are about half for England and about a quarter or a fifth for the continent, not to mention the fact that the original service, meal, theatre ticket, carriage hire, etc., is considerably cheaper.

Local transportation. Street car fare is usually two or three cents; cabs from 40 to 60 cents an hour, according to city, and upward for those of higher grade. In Paris and some other cities, the inclosed part of a car or omnibus costs double a seat on the roof. The latter is preferable in good weather for the sake of seeing interesting sights in passing, and in bad weather because the ventilation inside is nil. Walking is the most satisfactory way of getting in touch with the life of the people, but one must resist the temptation to overdo it. Incidentally, it may be suggested that shoes are about the only common article in which America surpasses Europe, either in quality or cheapness, and two or three pairs of shoes partly broken in, should be taken. However, counting time and trouble, expense of transportation and the American custom house inquisition, which is worse than for all western Europe put together, it scarcely pays to make ordinary purchases abroad.

Museums and points of interest. Speaking broadly, the best of these are free, but one should not grudge the sums of five to twenty-five cents charged for the maintenance of others for sight seers, nor the very reasonable tips expected by guides, especially if one counts the cost of being near them. For thorough study of a place, a Baedecker is indispensible. It is a poor economy, but very restful, to do a city without this guide book. Local guide books are sold in most cities and towns of resort. They make interesting souvenirs, but cost more in the long run than Baedeckers and are about as thorough as our local American city guides.

Actual estimates of expense. A fairly good rough rule is this: Add the round-trip cost to the sea port, to the round trip passage fare, add railroad fare for the actual route to be covered, at 2 cents a mile, add $5.00 a day, to include hotel bills at about $3.00 a day and incidentals, add whatever is to be spent for clothing and other purchases. This represents travel on a very comfortable, but unassuming scale. In any large city, one can get a room and meals for a dollar a day, equivalent to what would cost half as much again in America. such minor incidentals as car fare, tobacco and light refreshments, solid or liquid, costing about half the American price. This last estimate, of course, applies only to stays of a week or more in a place. In England, good second-class hotels and restaurants are about as expensive as in the States, though generally cleaner, while those of the first class, exclusive of those intended for the rich natives and Americans are approxmately on a three dollar a day scale, so that it is hardly worth while to attempt much economy in this way until the continent is reached.

